# The effect of socioeconomic status on postpartum depression: a parallel mediation model

**DOI:** 10.1186/s40359-025-02756-3

**Published:** 2025-05-06

**Authors:** Shi-Min Chen, Yan-Yang Qiao, Yu Zong

**Affiliations:** 1 Department of Psychology, Huaiyin Normal University, Huai’an, China; 2 The Public Administration Department, Tianjin Administration College, Tianjin, China; 3 School of Marxism, Xuzhou Medical University, Xuzhou, China

**Keywords:** Socioeconomic status, Postpartum depression, Needs, Postpartum mothers, Parallel mediation model

## Abstract

**Background:**

Previous studies have proposed that socioeconomic status (SES) can affect postpartum depression (PPD) through the mediators of satisfaction with material needs, healthcare, maternity leave, and postpartum social support. However, empirical data to validate these propositions has been lacking. This study aims to examine if all the four mediators can significantly mediate the effects of SES on PPD, as well as to compare the effect sizes of these mediators using a parallel mediation model.

**Methods:**

A total of 328 mothers within 1 year after childbirth completed the Socioeconomic Status Questionnaire, Met Material Needs Questionnaire, Inpatient Satisfaction with Care Questionnaire, Self-care Ability Scale for Puerperal Women, Maternity Leave Questionnaire, Questionnaire for Social Support during the Postpartum Period, and Beck Depression Inventory.

**Results:**

The level of depression in lower-SES mothers (M = 18.38, SD = 6.88) was significantly higher than that in medium-SES mothers (M = 14.54, SD = 8.58), which was significantly higher than that in higher-SES mothers (M = 10.61, SD = 8.25). SES significantly affected satisfaction with material needs, healthcare, maternity leave, and postpartum social support. Satisfaction with material needs and postpartum social support had a significant impact on PPD, and played a significant mediating role in the relationship between SES and PPD, with mediating effect sizes of 40.0% and 30.3%, respectively. In contrast, healthcare and maternity leave did not significantly influence PPD, nor did they significantly mediate the effect of SES on PPD.

**Conclusions:**

Postpartum social support and satisfaction with material needs are two important mediators between SES and PPD, whereas healthcare and maternity leave are two insignificant mediators in this relationship. More actions should be taken to reduce PPD in postpartum mothers, particularly those lower-SES mothers.

**Supplementary Information:**

The online version contains supplementary material available at 10.1186/s40359-025-02756-3.

## Introduction

The postpartum period is a challenging and difficult transition period for mothers. They experience many physical changes after giving birth, and shoulder the responsibility of taking care of their young babies. In addition, working women are often faced with work-family conflict. All kinds of pressures lead to women being highly susceptible to psychiatric disorders such as postpartum anxiety, postpartum depression (PPD), and postpartum bipolar disorder [1]. PPD refers to a depressive episode within one year after childbirth, and is commonly characterized as low mood, sadness, transient emotional instability, irritability, agitation and insomnia [2–4]. Meta-analyses have indicated that the global prevalence of PPD is 17% [4], and the prevalence in China is 14.7% [5]. The prevalence of PPD in China decreased from 17.1% in the first week to 8.5% in the sixth month postpartum, with significantly higher prevalence in underdeveloped areas compared to developed regions [5].

PPD has many adverse effects. It can lead to feelings of low mood, sadness, despair, and even suicidal ideation among mothers [6]. Furthermore, PPD can have negative effects on maternal breastfeeding [7], sensitivity to infant cues, interaction with the infant, which in turn can adversely impact the child’s physical health, motor skills, language development, cognitive development [8], as well as emotional well-being, behavior, attachment, and peer relationships [9, 10]. Additionally, postpartum depression can also affect family relationships, increase marital conflicts, and the risk of divorce [11, 12]. Therefore, early diagnosis and intervention for postpartum depression are of importance.

PPD is influenced by various factors, including physiological, psychological, familial, and sociocultural factors [13–16], with socioeconomic status (SES) being a particularly significant factor [2, 3, 17, 18]. SES refers to the position of individuals or families in the social stratum according to the material, human and social capitals they own [19–22]. Generally, material capital is assessed based on family assets and income; human capital is evaluated by educational attainment; and social capital is gauged through occupational categories, prestige, and social standing [20, 22].

Previous studies have shown that SES has a negative effect on PPD using t-tests or regression analyses [2, 3, 17, 18, 23]. This association can be understood through the lens of needs satisfaction, as postpartum mothers experience increased demands for material resources, healthcare, maternity leave, and postpartum social support, all while navigating the challenges of infant care and their own recovery. Higher SES appears to facilitate better fulfillment of these needs, thereby potentially mitigating PPD risk [2, 3, 17, 18, 23]. However, existing studies have primarily proposed mediating mechanisms theoretically, without empirical validation through collected data. Two critical research gaps remain unaddressed: (1) whether all four hypothesized mediators (satisfaction with material needs, healthcare, maternity leave, and postpartum social support) demonstrate significant mediating effects, and (2) what comparative effect sizes exist among these mediators. The current study aims to investigate these questions using a parallel mediation model, with the mediating hypotheses outlined as follows.

### Satisfaction with material needs

Following childbirth, both the mother and newborn require a variety of essential supplies, including food, clothing, daily necessities, and medications. Additional space may also be necessary to accommodate caregivers—such as grandparents or babysitters—who often assist in postpartum care. Furthermore, reliable transportation is crucial for managing tasks related to maternal and infant well-being. However, mothers with low SES frequently struggle to meet these needs, leaving them feeling overwhelmed, unsupported, and dissatisfied [2, 17, 18]. This lack of adequate resources contributes to an increased risk of PPD [24]. Therefore, the following hypothesis was presented.

Ha: Satisfaction with material needs would mediate the effect of SES on PPD.

### Healthcare

Women undergo significant physical changes during pregnancy, childbirth, and the postnatal period, which can lead to various complications [15]. And for babies, the first 28 days of life period constitute the most vulnerable time for a new-born’s survival [25]. Given these vulnerabilities, both mothers and infants require attentive healthcare. This healthcare demand encompasses two key aspects: (1) healthcare access, which refers to the availability of professional medical care for mothers and newborns, and (2) healthcare abilities, which involve the maternal knowledge and skills necessary for self-care and infant care. Mothers with higher SES benefit from superior professional healthcare when when there are health troubles with them or newborns [26]. Moreover, compared to mothers with low SES, those with higher SES have greater access to diverse resources for developing caregiving competencies, including instructional books, digital platforms, specialized training programs, and professional consultations [27]. These resources facilitate faster postpartum recovery, improve infant care practices, and foster a greater sense of control—all of which contribute to a reduced risk of PPD [28, 29]. Therefore, the hypothesis was proposed as below.

Hb: Healthcare would mediate the effect of SES on PPD.

### Maternity leave

Maternity leave refers to the legally guaranteed period of time off work for female employees during a woman’s pregnancy, delivery and baby care [30]. Mothers need a long time to recover after childbirth, and vulnerable new-born infants also need a long period to be cared for. Therefore, a certain period of maternity leave is very important. Mothers with higher educational attainment typically secure employment that provides comprehensive benefits, including extended paid maternity leave [31]. Additionally, those from economically advantaged households experience reduced financial constraints, allowing them to have a long maternity leave without rushing to work after childbirth [31]. Previous studies have shown that mothers with higher education and income enjoy longer paid maternity leave than their counterparts [31, 32]. A certain length of maternity leave helps reduce postpartum depression. In the United States and Australia, women who take more than 12 weeks of paid maternity leave report a significantly lower level of depression than women who take less than 12 weeks [31, 33]. In China, the prevalence of PPD is significantly higher in women who take a maternity leave of less than 98 days (14 weeks) than that in women who take a maternity leave of more than 98 days in China [32]. Therefore, the hypothesis was presented as follows.

Hc: Maternity leave would mediate the effect of SES on PPD.

### Postpartum social support

Mothers require substantial instrumental support to regain their health and care for their newborns after childbirth. Additionally, they often experience anxiety, depression, and irritability [1], making emotional support from others essential. Social exchange theory holds that social interaction is an exchange process [34]. People expect to maximize benefits and minimize costs in the process of exchange. If the exchange costs outweigh the benefits, people will adjust or terminate the exchange relationship. When the exchange costs are roughly equal to the benefits, the relationship will last [34]. Mothers with higher SES possess greater exchangeable resources, enabling them to access more comprehensive social support networks - including both instrumental and emotional support [35–37]. This robust support system facilitates successful adaptation during the postpartum transition and significantly mitigates the risk of PPD [38]. Therefore, the following hypothesis was proposed.

Hd: Postpartum social support would mediate the effect of SES on PPD.

The multiple mediation model is a unified statistical model into which multiple simple mediation models are analytically integrated [39]. Based on Ha, Hb, Hc and Hd, the following hypothetical parallel mediation model, a kind of multiple mediation model, was presented.

H1: SES would affect PPD through the mediators of satisfaction with material needs, healthcare, maternity leave and postpartum social support.

## Method

### Procedure

The ethical approval was obtained from the Academic Ethics Committee of Huaiyin Normal University before the survey was performed. The principles of voluntariness and confidentiality were underlined in the instruction section of the questionnaire. Participants were reminded that their participation was voluntary and that they could discontinue it at any time. They were assured that their responses would be kept confidential. Then they were asked to give informed consent at the beginning of the questionnaire.

The offline and online survey were both adopted. For offline survey, mothers, who held children in places such as parks, squares, and residential leisure areas, were investigated. For online survey, the questionnaire was uploaded to the website of “Questionnaire Star”, which was the most widely used survey website in China. Then, the electronic questionnaire was forwarded to those postpartum mothers and “Postpartum mothers” WeChat group by the social software of WeChat.

### Participants

A total of 401 questionnaires were obtained from 24 provinces in China. Invalid questionnaires were excluded according to two criteria. First, the child’s age, which was obtained by subtracting the date of birth from the test date, was more than 1 year. Second, there were inconsistent responses to lie-detection items (“There is a special person who cares about my feeling in my life” and “No one cares about my feeling in my life”). Three hundred twenty-eight valid questionnaires were obtained.

The age of the sample ranged from 21 to 45 years with a mean of 31.1 years (SD = 4.6). The sample consisted of 14 primary school graduates, 48 junior middle school graduates, 34 high school or technical secondary school graduates, 162 college graduates, 51 master’s graduates and 19 doctoral graduates. Fifty-one participants lived in rural areas, 64 in towns, 68 in counties, and 145 in urban districts. One hundred and seventy-three participants had single child, 147 had two children, and 8 had three children.

### Measures

#### SES

In this study, SES was assessed with four indicators, i.e., household assets, relative income level, educational level, and occupational category and position. Household assets have been evaluated by the ownership of equipment (washing machine, refrigerator, air conditioner, computer, water heater, telephone, TV, automobile, etc.) and the area of the flat in previous studies [40]. Because of the popularity of electrical appliances in China, the ownership of electrical appliances has become a nonsignificant indicator to show the differences in family economic conditions. Instead, the number and area of flats and the price of cars are currently two most important indicators to show the differences in family economic conditions in China. Therefore, the following two questions were used in this study to assess household assets. (1) What flat(s) do you and your husband own? The options were 0, 1 small flat with less than 90 square meters, 1 medium flat with 91–143 square meters, 1 large flat with more than 144 square meters, 2 flats, and 3 flats and above. These options were scored from 1 to 6. (2) What is the most expensive traffic vehicle in your family? The options were bicycle/electric bicycle/motorcycle, car with a price of less than 100,000 yuan, car with a price between 100,000 and 200,000 yuan, car with a price between 200,000 and 300,000 yuan, car with a price of more than 300,000 yuan. These choices were scored from 1 to 5.

Previous studies have shown that relative income level is a better indicator of financial status than actual income because the same monthly income indicates a different level of income in different regions [41]. For example, a monthly income of 5000 yuan can be at the medium income level in Chinese rural areas, but it is at a low level in metropolitan cities such as Beijing and Shanghai. Therefore, the relative income level was used in this study. Participants were asked to rate their monthly income level in their local area on a scale ranging from 1 (very low) to 5 (very high).

Educational level was assessed with the highest educational degree that the participant attained. The options were primary school graduate, junior middle school graduate, high school or technical secondary school graduate, college graduate, master’s graduate and doctoral graduate. These choices were scored from 1 to 6.

The Occupational Rank Questionnaire [42], integrating the occupational category and position, was used in this study. The occupations are classified into five ranks, with the sequences from low to high as follows: (1) unemployed, temporary workers, and physical laborers; (2) technical workers, self-employed workers and clerical staff; (3) junior professional and technical personnel, and junior managerial personnel; (4) middle-level professional and technical personnel, and middle-level management personnel; and (5) senior professional and technical personnel, and senior management personnel. These ranks were scored from 1 to 5.

Among the indicators of SES, a 6-point Likert scale was used to measure the area and number of flats and education level, whereas a 5-point Likert scale was used to evaluate the traffic vehicle, relative income level, occupational category and position. Because the scales of these indicators were different, they were standardized and their z-scores were aggregated as a composite SES score [43, 44].

#### Materials needs satisfaction

Materials needs satisfaction was assessed with the 6-item Met Material Needs Questionnaire, which was revised according to the Unmet Material Needs Subscale from the Economic Pressure Questionnaire [45]. Participants were asked to evaluate their satisfaction with the availability of food, clothing, housing, transportation, daily necessities and medication for themselves and their children after childbirth. Response were made on a scale ranging from 1 (unsatisfied very much) to 5 (satisfied very much). The model fit indices of confirmatory factor analysis all met the cut-off criteria (χ²/df = 2.10 ≤ 8.0, CFI = 0.966 ≧ 0.90, TLI = 0.955 ≧ 0.90, RMSEA = 0.041 ≤ 0.08), indicating that the model was acceptable. The reliability of the questionnaire in this study was Cronbach’s α = 0.805.

#### Healthcare

Healthcare was assessed with two indicators, i.e., satisfaction with medical services in hospital and postpartum mothers’ care knowledge and skills for themselves and their babies. Satisfaction with medical services in hospital was measured with 32-item Inpatient Satisfaction with Care Questionnaire developed by European Organization for Research and Treatment of Cancer [46], whose Chinese version was revised by Luo et al. (2014) [47]. This questionnaire assessses satisfaction with medical services in four ways: (1) satisfaction with doctors, including their technical skills, interpersonal skills, information provision, and availability; (2) satisfaction with nurses, including their technical skills, interpersonal skills, information provision, and availability; (3) satisfaction with services and care organization, including other hospital staff members’ interpersonal skills, wait times, information exchange, hospital access, hospital environment; and (4) overall satisfaction. Responses were made on a scale ranging from 1 (unsatisfied very much) to 5 (satisfied very much). The reliability of the questionnaire in this study was Cronbach’s α = 0.876.

Postpartum mother’s care knowledge and skills for themselves and their babies were measured with 42-item Self-care Ability Scale for Puerperal Women [48]. Postpartum mothers used the scale to self-rate their attitude, knowledge and ability to care for themselves and their babies. The options varied according to the questions. Responses were made on a scale ranging from 1 (completely disagree/do not know at all/cannot do it at all) to 5 (completely agree/fully know/can do it completely). The reliability of the questionnaire in this study was Cronbach’s α = 0.882.

Because the Inpatient Satisfaction with Care Questionnaire and Self-care Ability Scale for Puerperal Women both used a 5-point Likert scale, their average was directly used as the healthcare score.

#### Maternity leave

Maternity leave includes the length of maternity leave and maternity allowance. The original length of maternity leave (98 + 30 days) was further extended in each Chinese province in 2017 [49]. It has been extended to 128 days, 158 days, or 188 days in most provinces, even up to 1 year in some provinces. Therefore, the options were set as 0–30 days, 31–60 day, 61–90 days, 91–120 days, 121–150 days, 151–180 days, and 181 days above, which were scored from 1 to 7.

The maternity allowance is paid in terms of the average monthly salary in the year before the employee takes the leave according to the Social Insurance Law of the People’s Republic of China enacted in 2010. However, it is calculated with different concepts of salary in different organizations. The salary includes basic salary, allowance and so on in some organizations, but it only contains basic salary in others. In addition, the ratio of the basic salary to the total salary is variable in different organizations. Therefore, the proportion of the actual maternity allowance to the monthly total salary in the year before varies for different postpartum mothers. For the convenience of the participants, only eleven proportions were used in this study, that is, 0, 10%, 20%, 30%, 40%, 50%, 60%, 70%, 80%, 90%, and 100%. These options were scored from 1 to 11.

As for the indicators of maternity leave, a 7-point Likert scale was used to measure the length of maternity leave, while an 11-point Likert scale was used to measure the maternity allowance. Because the scales of these indicators were different, they were standardized and their z-scores were aggregated as a composite score of maternity leave.

#### Postpartum social support

Postpartum social support was assessed with 12-item Questionnaire for Social Support during the Postpartum Period [50]. This questionnaire measured the household activity support, informational support and emotional support for postpartum mothers during postpartum period. Responses were made on a 5-point scale indicating the frequency that the participants received social support after childbirth ranging from 1 (never) to 5 (always). The reliability of the questionnaire in this study was Cronbach’s α = 0.946.

#### PPD

PPD was assessed with 21-item Beck Depression Inventory-II [51], whose Chinese version was revised by Wang et al. (2011) [52]. The participants were asked to describe the severity of depressive symptom on a scale from 0 to 3 in the past week. The total score ranged from 0 to 63. The reliability of the questionnaire in this study was Cronbach’s α = 0.934.

### Data analysis

Relative criteria were used to determine the classes of SES in this study. Consistent with previous studies [43], plus and minus 1 SD was used as the cut-offs in this study. Descriptive statistics, correlational analysis and analysis of variance (ANOVA) were performed with the software of SPSS 26.0. Partial η² was used to assess the effect size of ANOVA in this study. When 0.01 ≤ partial η² < 0.059, the effect size is small; when 0.059 ≤ partial η² < 0.138, the effect size is medium; and when partial η² ≥ 0.138, the effect size is large [53, 54].

The mediation effect test was conducted by using structural equation modeling with the software of Mplus 7.4, which could control measurement errors and obtain more precise results than multiple linear regression [55, 56]. When the structural equation modeling was constructed, SES and maternity leave adopted observable variables because they were measured with one item of the total score; whereas the satisfaction with material needs, healthcare, postpartum social support and PPD used measurement models with latent variables. The bias-corrected nonparametric percentile bootstrap method was used to test the significance of the medication effect by drawing 2000 bootstrap samples with replacement from the full dataset (Shrout & Bolger, 2002). These samples were then used to construct a 95% or 90% confidence interval for the indirect effects. If this interval did not contain zero, the indirect effect was significant at *p* < 0.05. The mediation effect size is calculated according to the ratio of the indirect effect to the total effect.

## Results

### Descriptive statistics and bivariate correlations

The means, standard deviations and correlational coefficients among SES, satisfaction with material needs, healthcare, maternity leave, postpartum social support and PPD are shown in Table 1. The means of the standardized scores of SES and maternity leave were 0. There were significant positive correlations among SES, satisfaction with material needs, healthcare, maternity leave, and postpartum social support. PPD was negatively related to SES, satisfaction with material needs, healthcare, maternity leave, and postpartum social support.

**Table 1:** Means, standard deviations and correlational coefficients of main variables

Variables	1	2	3	4	5	6
1.SES	1					
2.SwMN	0.562**	1				
3.Healthcare	0.460**	0.448**	1			
4.Ma_leave	0.594**	0.371**	0.344**	1		
5.Soc_sup	0.429**	0.463**	0.693**	0.367**	1	
6.PPD	− 0.342**	− 0.449**	− 0.514**	− 0.331**	− 0.601**	1
M	0.00	3.07	3.31	0.00	3.14	14.51
SD	3.62	0.71	0.55	1.55	0.83	8.54

Notes: *N* = 328, SwMN = satisfaction with material needs, Ma_leave = maternity leave, Soc_sup = postpartum social support, PPD = postpartum depression, ** *p* < 0.01

### PPD in mothers with different SES

The mean total score of PPD was 14.51 (SD = 8.54) with the lowest total score of 0 and the highest total score of 51 in this study. Analysis of variance was performed on the levels of PPD in mothers with different SES. As shown in Table 2, the level of PPD in lower-SES mothers is significantly higher than that in medium-SES mothers, whereas the level of depression in medium-SES mothers is significantly higher than that in higher-SES mothers. The value of the partial η² (0.068) was greater than 0.059 with a medium effect size.

**Table 2:** Comparison among the levels of depression in mothers with different SES

Lower-SES(1)		Medium-SES(2)		Higher-SES(3)		F	Post hoc	Partial η²
M	SD	M	SD	M	SD			
18.38	6.88	14.54	8.58	10.61	8.25	11.78***	1 > 2 > 3	0.068

Note: ***, *p* < 0.001; >, significantly greater than

### Mediation effect test

**Fig. 1:**
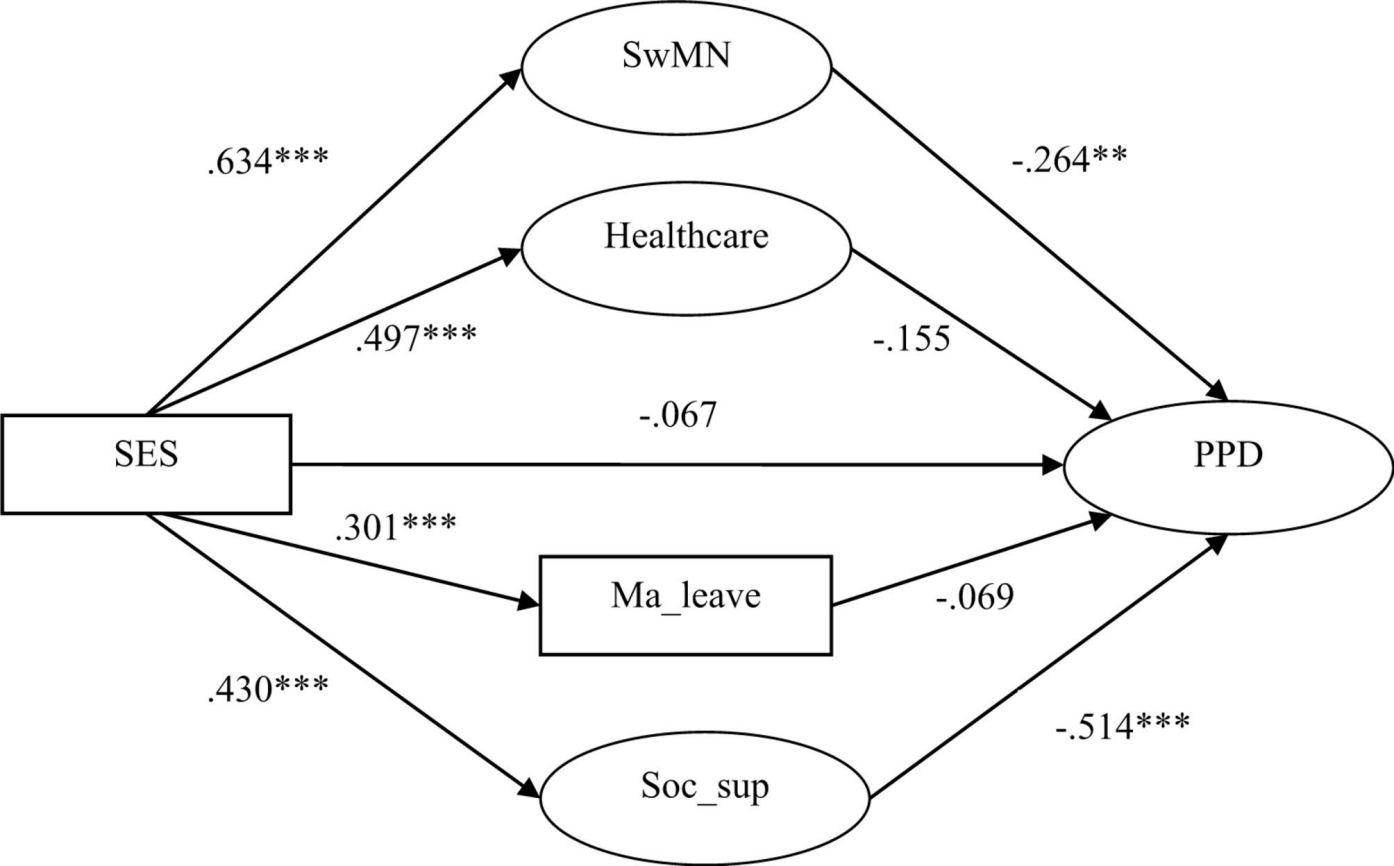
Parallel mediation model of the effect of SES on PPD. Notes: *N* = 328, SwMN = satisfaction with material needs, Ma_leave = maternity leave, Soc_sup = postpartum social support, PPD = postpartum depression, ** *p* < 0.01, *** *p* < 0.001

The structural equation modeling was constructed according to H1. The model fit indices all met the cut-off criteria (χ²/df = 2.41 ≤ 8.0, CFI = 0.918 ≥ 0.90, TLI = 0.907 ≥ 0.90, RMSEA = 0.065 ≤ 0.08), suggesting that the model was acceptable. The standardized coefficients are shown in Fig. 1. The bootstrap confidence intervals of mediation effect tests are indicated in Table 3.

**Table 3:** Confidence intervals of mediation effect tests and mediation effect sizes

Effect	Mediators	Eff_value	95%CI	Eff_size
Direct		-0.067	[-0.024, 0.062]	12.1%
Indirect	SwMN	-0.167	[-0.088, -0.005]	30.3%
	Healthcare	-0.077	[-0.049, 0.007]	13.9%
	Ma_leave	-0.021	[-0.015, 0.003]	3.8%
	Soc_sup	-0.221	[-0.089, -0.033]	40.0%
	Tot_ind	-0.486	[-0.180, -0.089]	87.9%
Total		-0.553		

Notes: *N* = 328, eff_value = effect values, eff_size = effect sizes, SwMN = satisfaction with material needs, Ma_leave = maternity leave, Soc_sup = postpartum social support, tot_ind = total indirect

The confidence intervals for the mediating effects of satisfaction with material needs and postpartum social support on the relationship between SES and PPD did not contain 0, indicating that both mediating effects were significant, with the mediating effect sizes of 30.3% and 40.0%, respectively. When comparing the mediating effect values of satisfaction with material needs and postpartum social support, the confidence interval for their difference was [-0.031, 0.017]. Since this confidence interval included 0, it indicated that there was no significant difference between the two mediating effects.

The confidence intervals for the mediating effects of healthcare and maternal leave on the relationship between SES and PPD contained 0, indicating that neither of the mediating effects was significant. The confidence intervals for the total mediating effects did not include 0, suggesting that the total mediating effect was significant, with a total mediating effect size of 87.9%.

Insert Fig. 1; Table 3 here.

## Discussion

### Effect of SES on PPD

This study aimed to examine the mediating effect of SES on PPD with a parallel mediation model. ANOVA showed that the lower the SES was, the higher the level of depression was among postpartum mothers (Table 3). Figure 1 shows that SES had a significant effect on satisfaction with material needs, maternity leave, healthcare, and postpartum social support in the first stage of the parallel mediation model; whereas the effects of satisfaction with material needs, maternity leave, healthcare and postpartum social support on PPD exhibited considerable variation in the second stage of the parallel mediation model. The mediating effects of these four variables between SES and PPD were analyzed as below.

SES exhibited the strongest standardized regression coefficient in predicting satisfaction with material needs (Fig. 1). For postpartum mothers, the needs for clothing, food, housing, transportation, daily necessities, and medical care were particularly pressing. The fulfillment of these material needs was most significantly influenced by SES. Moreover, the satisfaction with material needs demonstrated a significant negative predictive effect on PPD (Fig. 1), aligning with findings from prior research [24]. According to Maslow’s hierarchy of needs theory [57] and the hierarchy of needs theory in evolutionary psychology [58], material needs are the most fundamental and urgent for humans. The fulfillment of these needs can enhance an individual’s sense of well-being. In contrast, prolonged unmet material needs can lead to feelings of anxiety, tension, frustration, and helplessness, ultimately resulting in depression. The mediating effect test showed that satisfaction with materials needs significantly mediated the relationship between SES and PPD, thereby supporting Hypothesis Ha.

SES significantly influenced the healthcare access and abilities of postpartum mothers, which was consistent with the findings of previous studies [26, 27]. However, the impact of healthcare access and abilities on PPD was relatively small and did not reach a significant level, which contradicted the initial hypothesis. This may be associated with the widespread Chinese postpartum practice of Zuoyuezi (坐月子), commonly referred to as “doing the month”. The postpartum mothers stay at home for about one month after childbirth, during which family members take care of the mother and the newborn, passing on various caregiving methods [59]. Since postpartum mothers can receive care from family members, they are less influenced by the quality of medical services. Moreover, family members share childcare knowledge and practical skills with them—regardless of scientific validity, these contributions help mitigate postpartum anxiety and depression in postpartum mothers [60]. As a result, healthcare access and abilities play a relatively limited role in PPD. The mediating effect test revealed that healthcare did not significantly mediate the effect of SES on PPD. Thus, Hypothesis Hb was not supported.

SES had a significant effect on maternity leave, consistent with the findings of previous studies [31, 32]. However, the effect of maternity leave on PPD was not significant, which also contradicted the initial hypothesis. This could be attributed to the possibility that a more crucial factor affecting PPD may not be the actual maternity leave, but rather the satisfaction with maternity leave, which is defined as the gap between the actual and ideal maternity leave. Although some mothers desire an extended maternity leave to provide better care for their babies, others may prefer a shorter leave for various reasons. For instance, some mothers with low SES may wish to return to work sooner to boost their income. Additionally, mothers who prioritize career achievement may be eager to resume work to pursue further accomplishments. Furthermore, some mothers may fear being replaced if they take an extended leave. The mediating effect test revealed that maternity leave did not significant mediate the relationship between SES and PPD. Consequently, Hypothesis Hc was not supported.

As shown in Fig. 1, SES had a significant effect on postpartum social support, which aligned with the findings of prior research [35–37]. Moreover, postpartum social support had a significantly negative impact on PPD, which was also consistent with previous literature [38]. For postpartum mothers with higher education and income, they can receive more social support, especially from their mothers-in-law and biological mothers. In Chinese families, the relationship between mothers-in-law and daughters-in-law is distinct and noteworthy. This relationship is neither based on blood ties, like parent-child relationships, nor on love, as in marriage, but rather emerges from the important connections derived from these two core family ties. As a result, the mother-in-law and daughter-in-law relationship is significant and complex. The quality of this relationship plays a crucial role in the stability and happiness of the family. With the transformation of contemporary Chinese social structures and the evolution of values, particularly the significant improvement in women’s education levels and the normalization of their participation in the workforce, the traditional power dynamics within mother-in-law and daughter-in-law relationships have undergone fundamental changes. Modern mother-in-law and daughter-in-law relationships are gradually evolving from the traditional vertical structure of “respect and hierarchy” to more egalitarian relationships [61]. This new dynamic emphasizes mutual benefit and win-win outcomes. Postpartum mothers with higher SES are more likely to gain social support from their mothers-in-law, rather than experiencing the traditional patterns of estrangement, exclusion, or even conflict. Moreover, the survey indicates that as the economic conditions of grandparents and adult children improve, the likelihood of cohabitation decreases; conversely, poorer economic conditions lead to a higher likelihood of living together [62]. When postpartum mothers do not live with their mothers-in-law, the possibility of their biological mothers coming to provide postpartum care becomes feasible. Generally speaking, biological mothers are more likely to offer postpartum mothers attentive care and emotional support compared to mothers-in-law. These instrumental and emotional social support plays a crucial role in their physical recovery, childcare, and the reduction of PPD for postpartum mothers. The mediating effect test indicated that postpartum social support significantly mediated the relationship between SES on PPD, demonstrating the greatest mediating effect size. Therefore, Hypothesis Hd was supported.

In summary, SES significantly affected satisfaction with material needs, healthcare, maternity leave, and postpartum social support. Satisfaction with material needs and postpartum social support had a significant impact on PPD, and played a significant mediating role in the relationship between SES and PPD. In contrast, healthcare and maternity leave did not significantly influence PPD, nor did they significantly mediate the effect of SES on PPD. In other words, postpartum social support and satisfaction with material needs served as two important mediators between SES and PPD, whereas healthcare and maternal leave were two insignificant mediators in this relationship.

### Highlights

This study yielded the following highlights. Previous studies have theoretically suggested that satisfaction with material needs, healthcare, maternity leave, and postpartum social support might serve as mediators in the relationship between SES and PPD. This study utilized a parallel mediation model to validate the mediating roles of the four variables. The results indicated that SES significantly influenced satisfaction with material needs, healthcare, maternity leave, and postpartum social support. However, only satisfaction with material needs and postpartum social support were found to significantly affect PPD, serving as important mediators between SES and PPD. In contrast, healthcare and maternity leave did not have a significant effect on PPD, and did not act as effective mediators in this relationship. Unlike the speculative nature of earlier studies, this research provided empirical data that enhances understanding of how SES influences PPD in a more accurate and nuanced manner.

### Practical implications

This study provided guidance to reduce PPD in postpartum mothers, especially those lower-SES mothers. Firstly, for lower-SES postpartum mothers who meet specific policy criteria, particularly those who have given birth to multiples, direct living subsidies can be provided to help meet their material needs.

Secondly, we can help postpartum mothers to obtain more social support, as postpartum social support has the most important effect on PPD. Mothers from lower-SES mothers often experience limited social support [2, 18]. By assisting these mothers in improving their help-seeking skills, we can empower them to receive greater social support.

Third, postpartum mothers, especially rural postpartum mothers, can be assisted in improving their healthcare knowledge and skills. For many postpartum mothers, their healthcare knowledge and skills are often passed down through the experience of family elders, which may not be scientific enough [63]. Healthcare training can be provided to them to enhance their healthcare knowledge and skills.

Last but not the least, flexible regulations on the length of maternity leave should be established. According to the new regulations on maternity leave in 2022, most Chinese provinces and cities do not have flexible maternity leave regulations except Beijing and Fujian. The expected length of maternity leave should be investigated, and more flexible regulations should be established to better meet the needs of different mothers and families.

### Limitations and future directions

Despite its contributions, some limitations of this study should be acknowledged. The first limitation is the use of self-reported measures, which can be subject to the social desirability effect, potentially producing subjective and inaccurate responses [64, 65]. Future research should use other types of methods, such as other rating tools, to obtain more objective and accurate data. Second, the path coefficient of maternity leave on PPD was not significant in this study. Satisfaction with maternity leave may be a better predictor. Further studies can be performed on the relationships among SES, satisfaction with maternity leave and PPD. Finally, PPD is influenced, to some extent, by local customs and various other factors. The participants in this study were exclusively postpartum mothers in China. Are the conclusions drawn from this study universally applicable? Future research could investigate a more diverse sample in other countries and regions.

## Conclusion

The level of PPD in lower-SES mothers is significantly higher than that in higher-SES mothers. SES significantly impacts satisfaction with material needs, healthcare, maternity leave, and postpartum social support. Satisfaction with material needs and postpartum social support have a significant effect on PPD, and play a significant mediating role in the relationship between SES and PPD. In contrast, healthcare and maternity leave do not significantly influence PPD, nor do they significantly mediate the effect of SES on PPD. In other words, postpartum social support and satisfaction with material needs serve as two important mediators between SES and PPD, whereas healthcare and maternal leave are identified as two insignificant mediators in this relationship.

More actions should be taken to reduce PPD in postpartum mothers, especially those lower-SES mothers. These actions include offering living subsidies to postpartum mothers, assisting postpartum mothers in accessing to more social support, improving their healthcare knowledge and skills, and implementing flexible maternity leave regulations.

## Electronic supplementary material

Below is the link to the electronic supplementary material.

Supplementary Material 1

## Data Availability

Data is provided in the supplementary information file.

## Abbreviations

SES: Socioeconomic status
PPD: Postpartum depression
