# Correction: The effect of socioeconomic status on postpartum depression: a parallel mediation model

**DOI:** 10.1186/s40359-025-02915-6

**Published:** 2025-06-05

**Authors:** Shi-Min Chen, Yan-Yang Qiao, Yu Zong

**Affiliations:** 1 Department of Psychology, Huaiyin Normal University, Huai’an, China; 2 The Public Administration Department, Tianjin Administration College, Tianjin, China; 3 School of Marxism, Xuzhou Medical University, Xuzhou, China

**Correction: BMC Psychology (2025) 13:476**

10.1186/s40359-025-02756-3

Following publication of the original article, it came to the journal’s attention that the authors names were incorrectly ordered in the author list. The order has since been corrected and the correct order can be seen in this erratum. The journal thanks you for reading this erratum and apologizes for any inconvenience caused by this processing error.

